# Influence of Microgel and Interstitial Matrix Compositions on Granular Hydrogel Composite Properties

**DOI:** 10.1002/advs.202206117

**Published:** 2023-01-30

**Authors:** Victoria G. Muir, Shoshana Weintraub, Abhishek P. Dhand, Hooman Fallahi, Lin Han, Jason A. Burdick

**Affiliations:** ^1^ Department of Bioengineering School of Engineering and Applied Sciences University of Pennsylvania Philadelphia PA 19104 USA; ^2^ School of Biomedical Engineering Science and Health Systems Drexel University Philadelphia PA 19104 USA; ^3^ BioFrontiers Institute University of Colorado Boulder Boulder CO 80303 USA; ^4^ Department of Chemical and Biological Engineering College of Engineering and Applied Science University of Colorado Boulder Boulder CO 80303 USA

**Keywords:** 3D printing, biomaterials, granular, hyaluronic acid, hydrogels

## Abstract

Granular hydrogels are an emerging class of biomaterials formed by jamming hydrogel microparticles (i.e., microgels). These materials have many advantageous properties that can be tailored through microgel design and extent of packing. To enhance the range of properties, granular composites can be formed with a hydrogel interstitial matrix between the packed microgels, allowing for material flow and then stabilization after crosslinking. This approach allows for distinct compartments (i.e., microgels and interstitial space) with varied properties to engineer complex material behaviors. However, a thorough investigation of how the compositions and ratios of microgels and interstitial matrices influence material properties has not been performed. Herein, granular hydrogel composites are fabricated by combining fragmented hyaluronic acid (HA) microgels with interstitial matrices consisting of photocrosslinkable HA. Microgels of varying compressive moduli (10–70 kPa) are combined with interstitial matrices (0–30 vol.%) with compressive moduli varying from 2–120 kPa. Granular composite structure (confocal imaging), mechanics (local and bulk), flow behavior (rheology), and printability are thoroughly assessed. Lastly, variations in the interstitial matrix chemistry (covalent vs guest–host) and microgel degradability are investigated. Overall, this study describes the influence of granular composite composition on structure and mechanical properties of granular hydrogels towards informed designs for future applications.

## Graphical abstract

To expand the properties available in granular hydrogels for biomedical applications, an interstitial hydrogel matrix is introduced between jammed microgels based on hyaluronic acid. Granular hydrogel spatiotemporal properties (e.g., mechanics, structure) are influenced by the microgel mechanics and degradability, as well as the interstitial matrix volume, compressive mechanics, and bond strength.

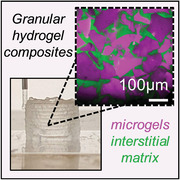

## 1 Introduction

Granular hydrogels are an exciting class of biomaterials that are being explored for many biomedical applications.^[^ ^1^, ^2^, ^3^ ^]^ To fabricate granular hydrogels, hydrogel microparticles, or “microgels,” are assembled into a granular media through microgel jamming.^[^ ^1^ ^]^ The granular structure imparts flowability as individual microgels slide past one another, while the microgel jamming allows the media to behave as a solid when stationary due to physical associations and frictional forces between the microgel grains.^[^ ^4^ ^]^ These properties make granular hydrogels particularly useful as injectable therapeutics or as extrudable inks for 3D printing applications.^[^ ^1^ ^]^ Further, due to the building‐block nature of granular hydrogels, multiple microgel populations can be mixed and patterned to further enhance the range of possible material properties.^[^ ^2^ ^]^

While granular hydrogels possess numerous advantages over traditional hydrogels, the weak physical and frictional interactions between microgels often results in low macroscale mechanical moduli and reduced hydrogel integrity.^[^ ^4^ ^]^ These limitations can be addressed through the introduction of interparticle crosslinking between microgels.^[^ ^4^ ^]^ For example, enzymatic crosslinking or photocrosslinking have been widely used to form covalent bonds between microgel particles to stabilize constructs.^[^ ^5^, ^6^, ^7^, ^8^, ^9^, ^10^ ^]^ Further, click chemistry reactions have been utilized to covalently link microgels by directly mixing microgel populations with complementary functional groups, such as tetrazine–norbornene and azide–alkyne pairs.^[^ ^11^, ^12^, ^13^, ^14^ ^]^ Reversible interparticle crosslinks have also been used, such as with guest–host bonds^[^ ^15^ ^]^ and ionic interactions.^[^ ^16^, ^17^ ^]^

While interparticle crosslinking allows for some improvement in mechanics and structural stability, there are some limitations to this approach, including the need to introduce unique chemistry to particles and mixing steps, geometric constraints that limit interparticle contact areas, and hindered mobility of reactive groups that are tethered to the microgel surfaces.^[^ ^4^ ^]^ An alternate approach is to utilize a crosslinkable interstitial matrix that resides between microgels, which still allows material flow but then supports secondary stabilization via interstitial matrix crosslinking.^[^ ^5^, ^18^, ^19^ ^]^ This composite structure with an interstitial matrix provides opportunities to design complex materials with multiple compartments (i.e., microgels and interstitial space) where various properties (e.g., degradation, mechanics) and content (e.g., encapsulated molecules, cells) can be varied. One potential limitation of using an interstitial matrix is the loss of porosity, which is often leveraged in granular hydrogels for cellular invasion.

Multiple granular hydrogel composites made from synthetic polymer systems have been investigated. For example, Amstad and colleagues fabricated polyacrylamide‐based granular hydrogel composites for 3D printing applications.^[^ ^5^, ^20^, ^21^ ^]^ In another example, Poletti and colleagues developed poly(ethylene glycol)‐based (PEG) granular hydrogel composites consisting of PEG microgels and an interstitial matrix of either cellulose^[^ ^22^ ^]^ or silk fibers^[^ ^19^ ^]^ for further reinforcement. It has also been shown that granular hydrogel composites can greatly improve print fidelity when compared to bulk hydrogels without microgels.^[^ ^23^ ^]^ Stimuli‐responsive granular hydrogel composites made from synthetic polymer systems have also been explored for biomedical applications.^[^ ^24^, ^25^ ^]^

Beyond synthetic systems, chemically modified biopolymers have also been used to fabricate granular hydrogel composites. Feng et al. developed a tissue‐adhesive granular hydrogel composite consisting of hyaluronic acid (HA) and gelatin microgels embedded in an interstitial matrix of HA modified with dopamine (HA‐DA) for 3D printing and tissue adhesion applications.^[^ ^26^ ^]^ In another example, Song et al. developed an extrusion printing ink consisting of gelatin microgels within a gelatin interstitial matrix.^[^ ^27^ ^]^ Such granular hydrogel composites have also been utilized for suspension bath printing. For example, Heo et al. developed a suspension bath consisting of gelatin microgels suspended in an oxidized alginate interstitial matrix.^[^ ^28^ ^]^ In another example, Molley et al. developed a gelatin granular hydrogel composite consisting of photocrosslinked gelatin‐methacrylamide (GelMA) microgels within a GelMA interstitial matrix.^[^ ^29^ ^]^ The GelMA granular hydrogel composite was used as a suspension bath for in vitro studies of tumor models and vascular channels.^[^ ^29^ ^]^

Granular hydrogel composites have much utility for biomedical applications; however, to date, a thorough understanding of how varying the compositions of microgels and interstitial matrices, as well as their relative ratios, remains limited. Herein, we investigated how the various design features of HA granular hydrogels influence composite properties, including variations in i) the ratios and crosslinking densities of microgels and interstitial matrix, ii) the type of crosslinks used in the interstitial matrix (i.e., covalent versus guest–host), and iii) the microgel degradability. Granular hydrogel composites were characterized through rheology, imaging (confocal imaging and 3D reconstruction), and mechanics (bulk compression and local moduli by atomic force microscopy [AFM]) and applied to 3D printing. Overall, this study investigates in depth how granular hydrogel composite composition influences granular hydrogel properties, where the various compartments guide complex biomaterials design for future applications.

## 2 Results and Discussion

### 2.1 Fabricating Granular Hydrogel Composites

Granular hydrogel composites consist of a granular hydrogel assembled from microgels, where the pore space between microgels is filled with a hydrogel interstitial matrix. In this study, microgels and interstitial matrices consisting entirely of photocrosslinkable hyaluronic acid (HA) were explored. As a first step toward fabrication, HA was modified with norbornene functional groups (NorHA) by anhydrous esterification of norbornene carboxylic acid to HA, as previously described.^[^ ^30^ ^]^ Norbornene degree of modification was determined to be 25% by ^1^H‐NMR (Figure S1, Supporting Information). The use of HA introduces a natural biopolymer found in the extracellular matrix (ECM) that has been widely used in biomedical applications, including multiple products that have translated to the clinic,^[^ ^31^, ^32^, ^33^ ^]^ in contrast to commonly used synthetic polymer systems within granular hydrogels, such as poly(ethylene glycol) (PEG)‐ and polyacrylamide (PAM)‐based networks. Compared to synthetic polymers, the use of HA introduces many desirable biomaterials properties, such as cytocompatibility and degradability.^[^ ^31^, ^34^ ^]^ However, chemically modified biopolymers, such as HA, tend to have overall weaker mechanical properties than synthetic polymer systems,^[^ ^35^ ^]^ such as PEG, PAM, and polyvinyl alcohol (PVA). This is important to consider when comparing the granular hydrogel composites explored herein with other granular hydrogel composite systems, which largely consist of synthetic polymer components.^[^ ^4^ ^]^ These comparisons and limitations are discussed throughout this work.

**Figure 1:**
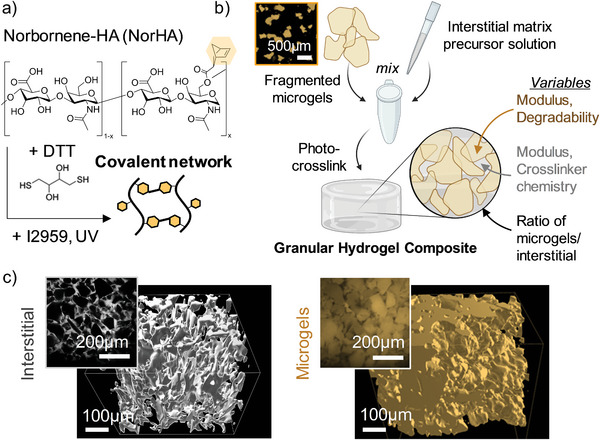
Fabricating granular hydrogel composites. a) Chemical structure of hyaluronic acid (HA) modified with norbornenes (NorHA) and schematic overview of the covalent crosslinking of NorHA with dithiothreitol (DTT) in the presence of Irgacure 2959 (I2959) photoinitiator and exposure to ultraviolet (UV) light. b) Schematic overview of granular hydrogel composite fabrication from fragmented microgels (inset: fluorescent images of fragmented NorHA microgels) and a photocrosslinkable interstitial matrix and variables investigated in the formulations related to the microgels (i.e., modulus, degradability), interstitial matrix (i.e., modulus, crosslinker chemistry), or the ratio of microgels to interstitial matrix. c) Representative 3D IMARIS reconstructions of confocal *z*‐stacks (left: interstitial region, silver; right: microgels, gold) of a granular hydrogel composite consisting of soft NorHA microgels (10 kPa) with 10% NorHA interstitial solution (120 kPa) added (insets: representative 2D slices).

To form hydrogels, NorHA was combined with dithiothreitol (DTT) and Irgacure 2959 (I2959) photoinitiator and crosslinked in the presence of light via a thiol‐ene reaction (**Figure** **1**a; Figure S2, Supporting Information). Five hydrogel formulations were investigated to vary NorHA hydrogel crosslink density, ranging from 0.9 to 4.5 wt% NorHA and (1–12) × 10^−3^ m DTT (Figure S2a, Supporting Information). All formulations photocrosslinked within seconds upon exposure to UV light (Figure S2b, Supporting Information) and resulted in bulk hydrogel compressive moduli ranging from 2 to 120 kPa (Figure S2c, Supporting Information). Across formulations, the concentration of free (i.e., unreacted) norbornenes after photocrosslinking (4 × 10^−3^ m) and the concentration of I2959 (0.05 wt%) were kept consistent.

Microgels were fabricated via extrusion‐based mechanical fragmentation, as previously described.^[^ ^36^, ^37^ ^]^ Hydrogels were photocrosslinked in bulk within a 3 mL syringe and subsequently extruded through a series of needles to yield extrusion fragmented microgels (Figure S3a, Supporting Information). Extrusion fragmentation was used due to the rapid, low‐cost, and scalable nature of the technique.^[^ ^36^, ^37^ ^]^ Further, in previous work, we demonstrated that microgels fabricated with extrusion fragmentation resulted in increased mechanical moduli when compared to granular hydrogels formed from spherical microgel populations.^[^ ^37^ ^]^ In addition, fabricating microgels by mechanical fragmentation can be applied to many polymer systems, such as cellulose,^[^ ^38^ ^]^ alginate,^[^ ^39^ ^]^ and polyacrylamide.^[^ ^25^ ^]^

To fabricate granular hydrogel composites, aqueous suspensions of microgels were first jammed by vacuum‐driven filtration, resulting in removal of the aqueous phase between microgels and maximum packing of the particles. Then, an aqueous solution of interstitial matrix precursor was added to the vacuum‐jammed microgels and thoroughly mixed to form a jammed suspension of microgels within an interstitial matrix precursor solution. This mixture was then exposed to ultraviolet (UV) light to photocrosslink the interstitial matrix and form a granular hydrogel composite (Figure 1b). This straightforward technique to fabricate granular hydrogel composites is amenable to polymer systems with multiple crosslinking chemistries, including photocrosslinking, thermal gelation, and temporally mediated click chemistries. The influence of five variables on granular hydrogel composite properties were investigated: i) microgel modulus, ii) interstitial matrix modulus, iii) the ratio of interstitial matrix/microgels, iv) interstitial matrix crosslinker chemistry, and v) microgel degradability (Figure 1b).

Both the interstitial matrix and microgel phases could be visualized using fluorescent confocal microscopy, and a 3D reconstruction of the granular hydrogel composite structure was obtained using IMARIS (Figure 1c; Video S1, Supporting Information). Taken together, the 2D confocal slices and 3D reconstructions demonstrate that the interstitial matrix was well‐mixed throughout the granular hydrogel composites (i.e., there were no pockets of microgel aggregates or interstitial matrix present). Thorough mixing to obtain a uniform distribution of particles is highly important for mechanical integrity in particle‐based composites.^[^ ^40^ ^]^

### 2.2 Influence of Microgel Modulus on Granular Hydrogel Composite Properties

**Figure 2:**
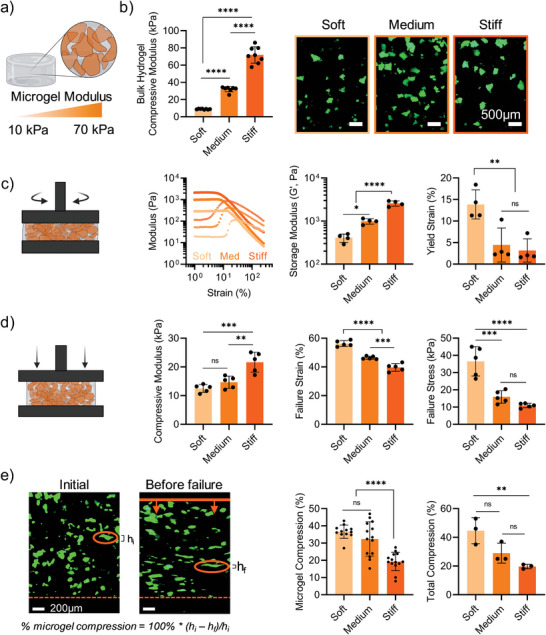
Influence of microgel modulus on granular hydrogel composite properties. Interstitial matrix is kept constant at 10% added by volume, with a modulus of 120 kPa. a) Schematic overview of granular hydrogel composites with varied microgel moduli. b) Bulk compressive moduli (*n* = 8) of hydrogels used to fabricate soft, medium, and stiff microgels (left), and representative images of fluorescently‐labeled microgels in suspension (right). c) Rheological characterization of granular hydrogel composite precursors prior to crosslinking of interstitial matrix, including representative strain sweeps (left, 1–250%), quantification of storage moduli (center, *G*′, Pa), and yield strain (right, %), *n* = 4. d) Compression testing of granular hydrogel composites (*n* = 5), including compressive moduli (left), failure strain (center), and failure stress (right). e) Visualization of granular hydrogel composites under compressive loading, including representative images of granular hydrogel composites containing fluorescently labelled soft microgels (1%) initially and immediately before failure during compressive loading (left), and quantified compression of individual microgels at failure (center, *n* = 14) and total compression of sample at failure (right, *n* = 3). Statistical analysis performed using a one‐way ANOVA. ns = no significance, *p < 0.05, **p < 0.01, ***p < 0.001, ****p < 0.0001.

We first sought to characterize the influence of microgel modulus on granular hydrogel composite properties (**Figure** **2**a), where microgels of varying moduli were fabricated via extrusion fragmentation. Extrusion fragmentation of soft (10 kPa), medium (30 kPa), and stiff (70 kPa) bulk hydrogels resulted in microgels that were jagged polygons in shape (Figure 2b) with diameters ranging from ≈10 to 300 µm (Figure S3b, Supporting Information). It is important to note that the distributions of microgel equivalent circular diameters, Feret's diameters, and aspect ratios were consistent across moduli (Figure S3b, Supporting Information). This suggests that microgel size and shape characteristics are a function of extrusion fragmentation conditions (e.g., needle size) and independent of bulk hydrogel modulus in this range (10–70 kPa).

It was previously found that the extrusion fragmentation technique as described is limited to bulk hydrogels with a compressive modulus less than ≈80 kPa.^[^ ^36^ ^]^ Above ≈80 kPa, excessive force is needed to push the hydrogel through the syringe and needle. Below ≈2 kPa, it is difficult to isolate the microgels by centrifugation. Thus, microgel moduli in the range of 10–70 kPa were explored in this study. Further, many soft physiological tissues possess mechanical moduli within this range of ≈1–100 kPa.^[^ ^41^ ^]^ For fragmenting hydrogels outside of this modulus range, alternate techniques could potentially be explored, such as fragmentation using a metal mesh, mortar and pestle, or blender.^[^ ^17^, ^23^, ^39^ ^]^

The degree of jamming and modulus of microgels have been shown to influence the rheological behavior of granular hydrogels.^[^ ^37^, ^42^ ^]^ Flow behavior is important to understand for multiple biomedical applications, including extrusion printing and injection into tissues.^[^ ^37^, ^42^, ^43^, ^44^ ^]^ Thus, we sought to thoroughly characterize the flow behavior of granular hydrogel composite precursors as a function of microgel modulus (Figure 2c). Granular hydrogel composite precursors were prepared with varied microgel modulus (soft, medium, and stiff) and a consistent interstitial matrix solution formulation (10 vol%, 120 kPa).

All compositions exhibited strain‐yielding, shear‐thinning, and self‐healing behaviors, as assessed by oscillatory shear rheology. As expected, storage moduli (*G*′, Pa) increased with increasing microgel modulus. The yield strain of granular hydrogel composite precursors was inversely proportional to the microgel modulus. For example, soft microgels had a significantly higher yield strain of ≈15%, compared to ≈5% for both medium and stiff microgels. A possible explanation for this trend is that softer microgels can withstand more deformation before yielding to flow due to the ability of the microgel matrix itself to deform.

The response of granular hydrogel biomaterials to compressive loading is important for multiple biomedical applications, including injection into load‐bearing tissues such as cartilage. Thus, we sought to thoroughly understand how microgel moduli influences the behavior of granular hydrogel composites under compressive loading (Figure 2d). In these studies, interstitial matrix precursor solutions were added to jammed microgels and photocrosslinked to form a stabilized granular hydrogel composite. We chose 10 vol% interstitial matrix with a modulus of 120 kPa across these studies, while varying microgel modulus (soft, medium, stiff).

The compressive moduli of granular hydrogel composites increased with increasing microgel modulus. For example, granular composites with stiff microgels had a 2‐fold higher compressive modulus than those with soft microgels. Interestingly, both failure strains and failure stresses were inversely proportional to microgel modulus. For example, granular composites consisting of soft microgels resulted in the highest failure strains (57%) and failure stresses (37 kPa). In contrast, granular hydrogel composites with stiff microgels had significantly lower failure strains (40%) and failure stresses (10 kPa).

A potential explanation for these trends is that, at low strains, granular hydrogel composites with stiff microgels can resist compressive loads. However, at intermediate strains, granular hydrogel composites with soft microgels can withstand higher loads than those with stiffer microgels, as soft microgels can more easily deform to distribute the load while the interstitial matrix holds the overall structure together prior to failure. These results are also consistent with the rheological behavior observed (Figure 2c), where soft microgels resulted in the highest yield strains. This is further evidence for the influence of soft microgels capable of enhanced deformation impacting the behavior of granular hydrogel composites.

Similar phenomena are observed in other particle‐based composite systems, which suggests that the observations in this study may be extended to granular composite systems made from other materials, even if the microgels and interstitial phases are made from distinct polymers. For example, toward applications in soft thermoconductive materials for flexible electronics, Tutika et al. demonstrated that including deformable liquid metal particles within a crosslinked silicon elastomer resulted in a decreased modulus and increased stretchability compared to silicon composites made from entirely rigid materials.^[^ ^45^ ^]^ In another example, Tang et al. demonstrated that incorporating soft rubber particles into an epoxy composite with rigid silica particles resulted in a decrease in overall moduli yet increases in fracture toughness and elongation at break,^[^ ^46^ ^]^ further highlighting how particles capable of greater deformation can enhance failure properties in composite materials. These properties and behaviors are important to consider for biomedical applications, where desired properties can be tailored based on material formulations. For instance, if a high compressive modulus is desired, granular hydrogel composites may be designed such that microgels have high moduli. However, if enhanced failure properties are desired, granular hydrogel composites may be engineered such that microgels are soft.

Due to the enhanced failure properties of granular hydrogel composites containing soft microgels, we hypothesized that softer microgels would support greater deformation during compressive loading than stiffer microgels, allowing for increased resistance to loading as well as increased failure strain and stress. To investigate this, we compressed granular hydrogel composite films (pseudo‐2D compression) where a fraction (1%) of microgels contained fluorescent dye (Figure 2e; Video S2, Supporting Information). Granular hydrogel composite films were compressed at a strain rate of 5 mm min^−1^ and monitored in real‐time using an epifluorescence microscope. Overall failure strains as well as compression (%) of individual microgels before failure were determined.

Granular hydrogel composite films with soft microgels had significantly higher bulk total compression before failure (≈45%) than systems with stiff microgels (≈20%), which was consistent with the bulk compression analysis (Figure 2d). Further, we were able to directly observe individual microgels deforming under compressive loading. Across microgel moduli, individual microgels compressed proportionally to the failure strain of the bulk samples (i.e., ≈40% for soft microgels, ≈20% for stiff microgels). On average, soft and medium microgels compressed significantly more than stiff microgels prior to failure. Taken together with the bulk compression behavior, these results are further evidence of softer microgels deforming more than stiffer microgels to improve the failure properties (i.e., strain and stress) of granular hydrogel composites.

### 2.3 Influence of Interstitial Matrix Modulus on Granular Hydrogel CompositeProperties

**Figure 3:**
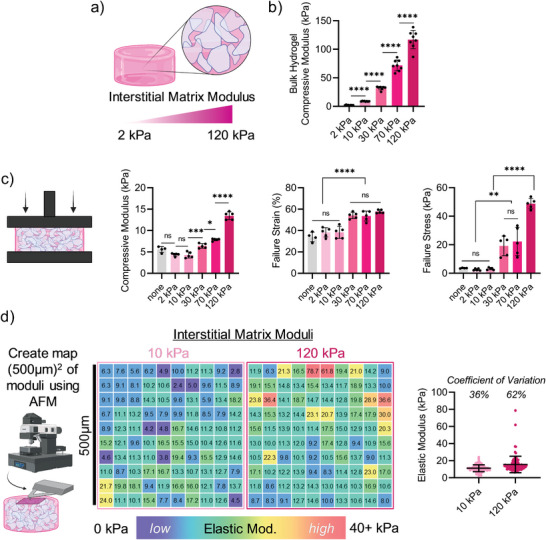
Influence of interstitial matrix modulus on granular hydrogel composite properties. a) Schematic overview of granular hydrogel composites with varied interstitial matrix moduli. b) Bulk compressive moduli (*n* = 8) of hydrogels used to fabricate interstitial matrices. c) Compression testing of granular hydrogel composites (*n* = 5), including compressive modulus (left), failure strain (center), and failure stress (right). “None” indicates inter‐particle photocrosslinking with no interstitial matrix present. Interstitial matrix kept consistent at 20% added by volume, and soft microgels used across composites. d) Schematic overview of atomic force microscopy (AFM) testing of the surface of granular hydrogel composites in a (500 µm)^2^ area (left), representative elastic moduli (kPa) heat maps (moduli measured every 50 µm in a (500 µm)^2^ area) for two granular hydrogel composite formulations where the interstitial matrix varied from 10 to 120 kPa (center), and quantified elastic moduli measurements (*n* = 100) across individual samples for the two granular hydrogel composite formulations. Interstitial matrix kept consistent at 10% added by volume, and soft microgels were used across composites. Statistical analysis performed using a one‐way ANOVA. ns = no significance, *p < 0.05, **p < 0.01, ***p < 0.001, ****p < 0.0001.

In addition to microgel modulus, we sought to characterize the impact of varying interstitial matrix modulus on granular hydrogel composite properties (**Figure** **3**a). In these studies, interstitial matrix precursor solutions were added to jammed microgels (soft) and photocrosslinked to form a stabilized granular hydrogel composite. We chose 20 vol% interstitial matrix with moduli from 2 to 120 kPa across these studies, to provide a wide structure–function relationship of the composites (Figure 3b). For comparison, granular hydrogels consisting of jammed microgels without interstitial matrix were investigated as a control, where microgels were covalently linked together via solely interparticle crosslinking. The interparticle photocrosslinking approach is one that is frequently used in granular hydrogel biomaterials to impart stability and enhanced mechanical moduli.^[^ ^7^, ^8^, ^47^, ^48^, ^49^ ^]^

The compressive moduli, failure strains, and failure stresses increased with increasing interstitial matrix modulus (Figure 3c). For example, the use of a 120 kPa interstitial matrix formulation resulted in a 3‐fold increase in compressive modulus and an 8‐fold increase in failure strain compared to a 2 kPa interstitial matrix modulus. These trends held true for granular hydrogel composites with both medium and stiff microgels as well (Figure S4, Supporting Information). Interestingly, the trends in microgel modulus being inversely proportional to failure strain and failure stress are largely consistent across interstitial matrix modulus. For example, when the interstitial matrix modulus was 2 kPa, soft microgels led to a failure strain of ≈39%, whereas stiff microgels led to a failure strain of 15%. Taken together, these results further highlight how both microgel and interstitial matrix moduli can be varied to tune granular hydrogel composite properties.

Most existing granular hydrogel systems rely on covalent interparticle crosslinks, rather than crosslinked interstitial matrices, to stabilize structures and enhance mechanical properties.^[^ ^4^ ^]^ As a control, granular hydrogels consisting of soft, medium, or stiff microgels were assembled with excess DTT and photoinitiator, and subsequently photocrosslinked to fabricate granular hydrogels with interparticle bonds yet no interstitial matrix (“none”). Importantly, the addition of interstitial matrix significantly increased failure strains and failure stresses across microgel moduli, compared to granular hydrogels with solely interparticle crosslinks (Figure 3b; Figure S4, Supporting Information). In particular, the addition of the stiffest interstitial matrix (120 kPa) resulted in 13‐fold, 10‐fold, and 5‐fold increases in failure stresses for soft, medium, and stiff microgels, respectively, compared to controls with interparticle crosslinking but without an interstitial matrix. These significant enhancements in mechanical integrity further highlight the need for interstitial matrices and granular hydrogel composites to widen the scope of potential mechanical behaviors of biomedical granular hydrogels.

To further characterize the local properties of granular hydrogel composites, AFM‐based nanoindentation was used to probe local mechanical properties. Comparison of elastic moduli measured in bulk by DMA or locally by AFM for the bulk hydrogel compositions (10 or 120 kPa) are shown in Figure S5 (Supporting Information). For soft bulk hydrogels, there was no significant difference between moduli measured by AFM or DMA; however, for the stiff bulk hydrogels, moduli determined by AFM were significantly lower than moduli determined by DMA. This is possibly due to incomplete crosslinking at the bulk hydrogel surface due to oxygen exposure (despite covering with a glass cover slip). In addition, the lower modulus for the stiff interstitial matrix may be due to the small dilution that occurs during mixing of the granular hydrogel composite precursor components, which is discussed further in Section 2.4. However, elastic moduli measured by AFM for the stiff bulk hydrogel (≈70 kPa) was still significantly higher than that of the soft bulk hydrogel (≈8 kPa), and thus local differences in moduli in granular hydrogel composites may be observed by AFM.

Granular hydrogel composites containing soft microgels, 10% interstitial matrix by volume, and either 10 or 120 kPa interstitial matrix compositions were evaluated by AFM (Figure 3d). A total of 100 measurements were sampled in a (500 µm)^2^ area to create a representative local map of elastic moduli in the granular hydrogel composite samples (Figure 3d). For granular hydrogel composites containing both soft microgels and soft interstitial matrix, elastic moduli were consistently around ≈10 kPa across the map, with a coefficient of variation of 36%. For granular hydrogel composites containing soft microgels and stiff interstitial matrix, while many regions had an elastic modulus around ≈10 kPa, there were pockets of regions with significantly higher local elastic moduli of 20–80 kPa, likely representative of the stiff interstitial matrix regions. Overall, granular hydrogel composites consisting of soft microgels with stiff interstitial matrices had a higher coefficient of variation (62%) for local elastic moduli. This further highlights the tunability of granular hydrogel composite systems, where local moduli can be varied at the microscale to enhance material properties.

In this study, a polystyrene sphere tip with radius *R* ≈ 5 µm was used as an AFM tip with a maximum indentation depth of 200–400 nm, yielding a tip‐sample contact radius of ≈1.4–2.0 µm. This is important to note, considering the feature size of the granular hydrogel composite, where microgels range in size from ≈10 to 400 µm and interstitial pockets range from ≈1 to 100 µm in diameter. The tip‐sample contact radius being one order of magnitude smaller than the size of the microgels and interstitial matrix features, and thus, could detect the spatial heterogeneity within and between these microdomains. When indenting throughout the 500 µm × 500 µm region, it is possible that a single indentation could probe an overlapping region of microgel and interstitial space. Molley et al. also observed a wide heterogeneity in stiffness measurements when using AFM‐nanoindentation to characterize GelMA‐based granular hydrogel composites.^[^ ^29^ ^]^ In their system, stiff GelMA microgels were crosslinked within a soft GelMA interstitial matrix. They directly indented two regions of interstitial space and two regions of microgel space, observing a 5‐fold increase in local stiffness in microgel regions compared to interstitial regions.^[^ ^29^ ^]^ They described that, in granular hydrogel composite systems, the interstitial matrix precursor not only fills the interstitial space, but also coats the microgel surface, which results in local heterogeneities in stiffness measurements such as those observed by AFM.^[^ ^29^ ^]^ Taken together, this highlights some challenges with probing local moduli by AFM in granular hydrogel composites, yet demonstrates that differences and heterogeneity in microgel and interstitial phase moduli can be observed.

### 2.4 Influence of Microgel/Interstitial Matrix Ratio on Granular Hydrogel Composite Properties

**Figure 4:**
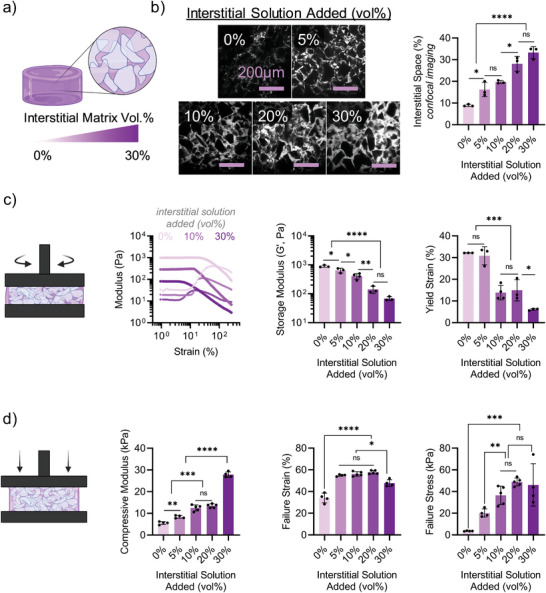
Influence of interstitial matrix/microgel ratio on granular hydrogel composite properties. Interstitial matrix (120 kPa) and microgel (soft) moduli kept consistent across granular hydrogel composites. a) Schematic overview of granular hydrogel composites with varied interstitial matrix volume fractions. b) Representative confocal image slices of granular hydrogel composites showing microgels (black) and pores (white) (left), and quantified interstitial space (%) as a function of interstitial solution added (vol %) (right, *n* = 3). Scale bar = 200 µm. c) Rheological characterization of granular hydrogel composite precursors prior to crosslinking of interstitial matrix, including representative strain sweeps (left, 1–250%), quantification of storage moduli (center, *G*′, Pa), and yield strain (right, %), *n* ≥ 3. d) Compression testing of granular hydrogel composites (with interstitial matrix crosslinked), including compressive modulus (left), failure strain (center), and failure stress (right), *n* ≥ 4. Statistical analysis performed using a one‐way ANOVA. ns = no significance, *p < 0.05, **p < 0.01, ***p < 0.001, ****p < 0.0001.

In addition to changing the material formulations for both microgels and interstitial matrix, we also sought to investigate the influence of interstitial volume fraction (0–30%) on granular hydrogel composite properties (**Figure** **4**a). The microgel modulus (soft) and interstitial matrix modulus (120 kPa) were kept consistent across formulations. To visualize the structure of granular hydrogel composites, a trace amount of FITC‐dextran (2MDa) was added to the interstitial matrix precursor solution prior to granular hydrogel composite fabrication. Confocal microscopy was used to visualize 2D cross sections (500 × 500 µm) of the granular hydrogel composites (Figure 4b). The amount of observed interstitial space was quantified as a function of interstitial solution added (5–30%). The granular hydrogel composite structures were visualized for formulations with medium and stiff microgels as well (Figure S6, Supporting Information).

Across all groups, the observed amount of interstitial matrix was slightly higher (≈5–10%) than the volume of interstitial solution added (Figure 4b). This is likely due to the presence of ≈5–10% void space within vacuum‐jammed microgels prior to the addition of interstitial matrix. However, for a given volume of interstitial solution added, the observed interstitial space was consistent across microgel moduli. These observations are important to understand when comparing mechanical properties as a function of microgel and interstitial matrix moduli in subsequent studies. Overall, the amount of interstitial matrix precursor solution added proportionally led to an increase in interstitial space (%), as others have reported as well.^[^ ^50^ ^]^

The existence of a small amount of void space (≈5–10%) in jammed microgels without interstitial solution is important to consider for composite formation. This results in some dilution of the interstitial matrix solution when added to microgels, which may slightly lower the interstitial matrix modulus compared to the bulk hydrogel formulation. In previously reported granular hydrogel composite systems, microgels were jammed in interstitial solution by centrifugation,^[^ ^5^, ^20^, ^42^, ^51^ ^]^ which also diluted the interstitial matrix due to the hydrated void spaces prior to the addition of interstitial matrix precursor. Jamming by vacuum‐driven filtration has been shown to result in more removal of the interstitial space than centrifugation;^[^ ^42^ ^]^ thus, dilution is likely less prevalent herein than in centrifuge‐packed granular hydrogel composites. Further, in this study, since there was no significant difference in void space of jammed microgels across microgel moduli, the impact of dilution will be consistent across a given interstitial solution volume percent. Lastly, it is important to note that as an alternative approach, dried microgels could be added to an interstitial precursor solution to form a granular hydrogel composite upon rehydration, which may overcome the impact of interstitial matrix dilution.^[^ ^18^, ^19^, ^21^, ^22^ ^]^ In this approach, drying and rehydration of microgels would have to be fully characterized to understand the impact on mechanical properties.

We also sought to characterize the flow behavior of granular hydrogel composite precursors as a function of the volume of interstitial space. As expected, storage moduli (*G*′, Pa) increased with decreasing interstitial volume (i.e., increased degree of jamming) (Figure 4c). The amount of interstitial solution added resulted in consistent decreases in the storage modulus across the various microgel moduli (Figure 4c; Figure S7, Supporting Information). For example, the addition of 10%, 20%, or 30% interstitial solution by volume resulted in a ≈50%, ≈75%, or ≈90% decrease in storage moduli, respectively, compared to no (0%) interstitial solution added (i.e., maximum packing) for soft, medium, and stiff microgels. For soft and medium microgels, the addition of a small amount (5%) of interstitial solution resulted in a ≈25% decrease in storage moduli. However, for stiff microgels, there was no significance decrease in storage modulus upon the addition of 5% interstitial solution. Further, the yield strain of granular hydrogel composite precursors was generally inversely proportional to the volume of interstitial solution.

In addition, we sought to investigate the influence of interstitial volume fraction on composite compressive moduli by varying the amount of interstitial solution added (Figure 4d). Increasing the amount of interstitial matrix resulted in an increase in both compressive moduli and failure stresses. These trends were true across granular hydrogel composites with either medium or stiff microgels as well (Figure S7, Supporting Information). Interestingly, compared to controls without any interstitial matrix, failure strains increased upon the addition of 5% interstitial matrix by volume, though it did not significantly change with the amount of interstitial matrix added (5–30%). For a given interstitial matrix vol%, the soft microgels yielded the highest failure strains and failure stresses, and the stiff microgels yielded the highest compressive moduli. Overall, these results demonstrate how changes in the interstitial matrix volume fraction can be used to further tune granular hydrogel composite properties.

The HA granular hydrogel composites in this study had both compressive moduli and failure stresses ranging from 2 to 60 kPa as a function of microgel modulus, interstitial matrix modulus, and interstitial matrix volume percent. This range of compressive moduli is on the higher end of what has been previously reported for biopolymer granular hydrogel systems crosslinked with click chemistry where the compressive moduli were ≈5 kPa or less.^[^ ^52^, ^53^, ^54^, ^55^ ^]^ A possible explanation for the increased moduli observed herein is the use of fragmented particles, which can interlock and enhance mechanical integrity compared to similar spherical microgel systems.^[^ ^37^ ^]^ It is also important to note that those previously reported studies used biopolymer concentrations of 7–10 wt%,^[^ ^52^, ^53^, ^55^ ^]^ whereas the granular hydrogel composites used in this study use an HA concentration of 4.5 wt% or less yet possessed higher mechanical moduli.

Importantly, granular hydrogels and composites have been fabricated with higher mechanical moduli, though it is important to note differences between these systems and the one described here. For example, granular hydrogels fabricated from GelMA with covalent interparticle interactions have been fabricated with reported compressive moduli of ≈50–100 kPa.^[^ ^48^, ^56^ ^]^ This system relied on microfluidic devices to fabricate spherical microgels from photocrosslinkable GelMA. In addition, this granular hydrogel formulation could withstand tensile testing, with a tensile modulus of ≈30 kPa.^[^ ^48^ ^]^ However, it is important to note that this system used 20 wt% GelMA, which is 6–20 fold times the concentrations used in this work. In granular hydrogel composites consisting of synthetic polymer components, significantly higher compressive and tensile moduli have been reported.^[^ ^4^ ^]^ For example, Hirsch et al. created a tough granular hydrogel composite consisting of PAMPS microgels in a PAM interstitial matrix.^[^ ^5^ ^]^ Polymer concentrations of 15–30 wt% were used, resulting in granular hydrogel composites with tensile moduli on the range of 20–400 kPa.^[^ ^5^ ^]^ In another example, Takahashi et al. created a tough adhesive particle hydrogel consisting of PAMPS microgels in a PAM matrix for surface coating applications.^[^ ^18^ ^]^ The resulting particle hydrogel had a maximum fracture stress of 2.4 MPa in tension.^[^ ^18^ ^]^

These studies highlight an important aspect of particle‐based composite systems – the impact of particle‐interstitial matrix adhesion on granular composite properties.^[^ ^40^, ^57^ ^]^ In particle composite systems, the failure properties often depend on the strength of the anchorage between the particles and the interstitial binder.^[^ ^40^, ^57^ ^]^ In the synthetic granular hydrogel systems described above,^[^ ^5^, ^18^ ^]^ microgels wre soaked in photocrosslinkable monomers, and a continuous polymer matrix permeates both the microgels and the interstitial space. This approach, combined with the significantly higher concentrations of polymers used, likely strengthens the anchorage between microgels and interstitial space, leading to higher mechanical moduli. However, creating a permeating network will likely significantly alter the properties within the microgels, making it challenging to maintain compartments with distinct mechanical moduli in the granular hydrogel composite structure.

In contrast, the HA granular hydrogel composite explored in this work likely does not contain a permeating interstitial matrix, making it distinct from these other granular composite systems. This may lead to weaker anchorage points between the microgel and interstitial space, though compartments with distinct moduli may be maintained. In future directions, concentrations of free norbornene at the microgel surface could be increased to improve anchorage to the interstitial matrix and potentially enhance mechanical moduli and failure properties. Further, future work may explore how using NorHA microgels with a distinctly different polymer system in the interstitial matrix, such as polyacrylamide, may impact mechanical properties.

### 2.5 Extrusion Printing with Granular Hydrogel Composites

**Figure 5:**
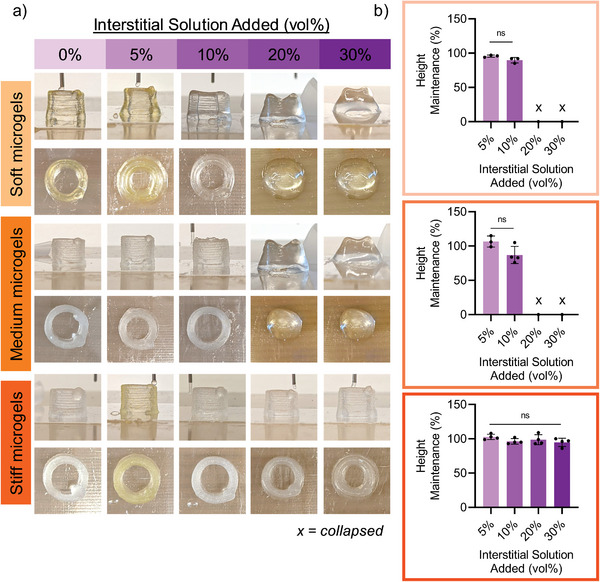
Extrusion printing with granular hydrogel composites. Interstitial matrix modulus (120 kPa) kept consistent across granular hydrogel composites. Left: Representative images (side and top views) of extrusion printed hollow cylinders (1 cm tall, 1 cm diameter) made from granular hydrogel composite precursor immediately after deposition for various microgel moduli (soft, medium, stiff) and interstitial solution volume percent (0–30%). Right: Quantification of height maintenance for granular hydrogel composite printed structures. An “x” indicates that the structure fully collapsed. Statistical analysis performed using a one‐way ANOVA. *n* ≥ 3, ns = no significance.

Granular hydrogels have been explored as extrusion printing inks due to their shear‐thinning and self‐healing behavior.^[^ ^6^, ^7^, ^43^, ^44^ ^]^ Further, the granular structure can impart immediate stability upon deposition even prior to post‐crosslinking and post‐processing steps, creating the potential for a simplified and straightforward printing process. We sought to characterize the printability of granular hydrogel composite precursors as a function of both varied microgel modulus (soft, medium, stiff) and varied amounts of interstitial solution (0–30%) (**Figure** **5**). To assess printability, we extrusion printed hollow cylinders (height: 1 cm, diameter: 1 cm, filament width: 2 mm) and observed their structures immediately after ink deposition by recording cylinder height (Figure 5a).

The successful printing of the various inks was dependent on the microgel modulus and the amount of interstitial matrix. All granular hydrogels without interstitial matrix were printable, due to the high jamming of the microgels, and all maintained their cylindrical shapes after printing. When the interstitial matrix was increased, the printing success decreased for both the soft and medium microgel moduli, with collapse of the structures with greater than 20 vol% of interstitial matrix added (Figure 5a). This indicates that the interstitial matrix decreases the jamming of the microgels, which is evident from the rheological characterizations with a reduction in both the storage moduli and yield strains for these formulations (Figure 4c; Figure S7, Supporting Information). It is interesting that the transition was abrupt from 10 to 20 vol%, which could relate to the level of interstitial porosity and the extent of packing of the microgels (Figure 4b). However, hollow cylinders printed with granular hydrogel composite precursors containing stiff microgels resulted in stable structures across all amounts of interstitial volume (0‐30%), further indicating the high structural stability imparted from the stiff microgels themselves in the granular hydrogel composite precursor inks. Increasing structural stability with increased microgel modulus has been observed in other granular hydrogels systems. For instance, Xin et al. observed that increased PEG microgel modulus resulted in increased structural stability in hollow cylinder structures printed with granular hydrogels.^[^ ^43^ ^]^

When designing hydrogel‐based extrusion printing inks, the storage modulus (*G*′) is often used as an indicator of extrusion printability, where higher storage modulus in a shear‐thinning and self‐healing ink leads to increased print stability.^[^ ^58^, ^59^ ^]^ It is interesting to note that granular hydrogel composites with stiff microgels and 30% interstitial matrix had a *G*′ of ≈320 Pa, whereas granular hydrogel composites with medium microgels and 20% interstitial matrix had a higher *G*′ of ≈580 Pa (Figure 4c; Figure S7, Supporting Information). The former resulted in a stable printed structure, yet the latter collapsed (Figure 5b), despite having a *G*′ nearly twice as high. This further highlights the unique material aspects of a granular hydrogel composites, where the granular structure significantly influences outcomes. Thus, it is important to consider the fundamentals of both hydrogel and granular material rheological behaviors when designing granular composite biomaterials.

To overcome structural collapse, granular hydrogel composite precursors with > 20% by volume interstitial and soft or medium microgels were exposed to UV light during the ink deposition (Figure S8, Supporting Information). This allowed for photocrosslinking of the interstitial matrix while the ink was being deposited, thus increasing structural stability. For medium microgels, photocrosslinking during ink deposition completely overcame collapsing. However, for soft microgels, structural stability was only slightly improved, and structures still exhibited partial collapse. Thus, for softer microgels, additional printing considerations such as light intensity, as well as print and extrusion speeds, may need to be tuned to improve structural stability of the printed constructs.

### 2.6 Introducing Guest–Host Crosslinking into Granular Hydrogel Composite Interstitial Matrices

Covalent crosslinking mechanisms, such as the thiol‐ene radical addition between NorHA and DTT, result in hydrogels with high mechanical moduli and stability. However, covalent bonds lack dynamic behavior. In contrast, guest–host interactions can be used to form adaptable and reversible crosslinks, where guest and host functional groups are used to assemble a hydrogel via reversible hydrophobic interactions.^[^ ^31^, ^60^ ^]^ Guest–host chemistry has been used toward biomaterials for drug delivery,^[^ ^61^ ^]^ tissue repair,^[^ ^51^ ^]^ and 3D printing.^[^ ^62^ ^]^ A common guest–host crosslinking system used to form self‐healing and shear‐thinning hydrogels uses adamantane (Ad, guest) and *β*‐cyclodextrin (CD, host) guest–host interactions, where Ad‐modified and CD‐modified polymers are mixed to form an injectable bulk hydrogel.^[^ ^63^, ^64^ ^]^ This crosslinking system has been translated to granular hydrogels using interparticle crosslinking.^[^ ^15^, ^51^ ^]^ For example, Widener et al. combined PEG microgels functionalized with either Ad or CD groups to form a granular hydrogel with physical guest–host interparticle crosslinks.^[^ ^15^ ^]^ The resulting system was shear‐thinning and self‐healing, and had enhanced structural stability compared to granular hydrogels without interparticle crosslinking.

Recent advances in guest–host networks have included systems where light is used to introduce the guest–host crosslinking via thiol‐ene reactions, which would provide a similar crosslinking methodology as in the systems described earlier in this work. For example, Hui et al. combined NorHA, CD‐modified HA (CDHA), and monothiolated adamantane (AD‐thiol) to create a photocrosslinkable guest–host hydrogel for spatiotemporal control over crosslink formation.^[^ ^65^ ^]^ In another example, Dhand et al. fabricated a one‐pot photocrosslinkable interpenetrating network (IPN) hydrogel by combining methacrylated HA (MeHA), NorHA, CD‐thiol, and AD‐thiol for additive manufacturing and biomedical applications.^[^ ^66^ ^]^

**Figure 6:**
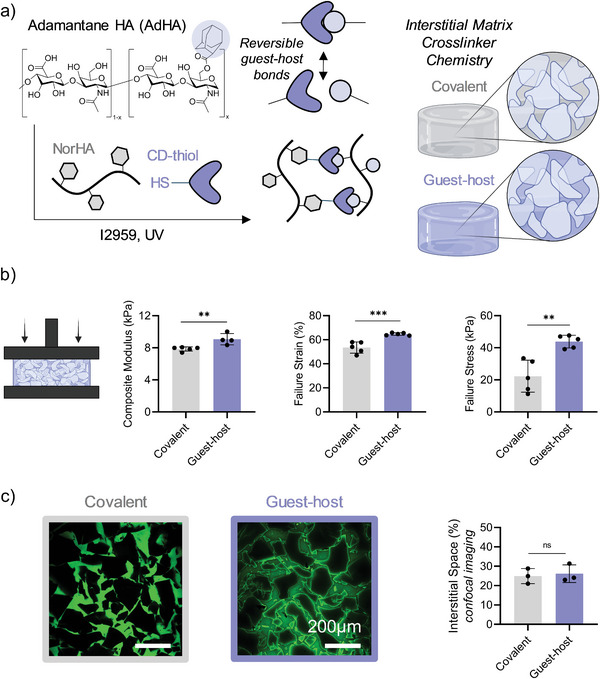
Influence of interstitial matrix crosslinker chemistry on granular hydrogel composite properties. Microgel modulus (soft, 10 kPa), total concentration of norbornene groups (28 × 10^−3^ m) in interstitial matrix, and amount of consumed norbornene groups (86%) in interstitial matrix kept consistent across granular hydrogel composites. a) Schematic depicting the chemical structure of adamantane‐modified HA (AdHA), and combination with NorHA, thiolated *β*‐cyclodextrin (CD‐thiol), I2959, and UV light to form a photocrosslinkable guest–host hydrogel with reversible guest (Ad) and host (CD) bonds (left). Schematic of granular hydrogel composites with either covalent or guest–host crosslinkers within the interstitial matrices (right). b) Quantified compressive moduli (left), failure strain (center), and failure stress (right) of granular hydrogel composites with either covalent or guest–host crosslinkers within the interstitial matrices (*n* = 5). c) Representative confocal image slices of granular hydrogel composites with either covalent or guest–host crosslinkers within the interstitial matrices showing microgels (black) and pores (green) (left) and quantified interstitial space (%) as a function of crosslinkers within interstitial matrices (right, *n* = 3). Scale bar = 200 µm. Statistical analysis performed using a one‐way ANOVA. **p < 0.01, ***p < 0.001.

Herein, a photocrosslinkable guest–host hydrogel was used as an interstitial matrix to fabricate granular hydrogel guest–host composites (**Figure** **6**a). NorHA was combined with adamantane‐modified HA (AdHA, ≈20% degree of modification [Figure S9, Supporting Information]), thiolated *β*‐cyclodextrin (CD‐thiol), and I2959 photoinitiator (Figure 6a). The total concentration of norbornene functional groups, as well as the degree of norbornene groups consumed by thiols in the thiol‐ene radical addition, was kept consistent between guest–host hydrogels and the stiffest covalent interstitial matrix explored herein (Figure S10a, Supporting Information). Upon exposure to UV light, a bulk guest–host hydrogel was formed by thiol‐ene radical addition between CD‐thiol and NorHA. In the presence of AdHA, the resulting system formed a photocrosslinkable guest–host bulk hydrogel with reversible crosslinks due to the dynamic guest–host interactions (Figure S10b, Supporting Information). Guest–host bulk hydrogels had a ≈2‐fold lower compressive modulus (≈50 kPa) compared to the covalent bulk hydrogel (≈120 kPa) (Figure S10c, Supporting Information).

Granular hydrogel guest–host composites were fabricated such that microgel modulus (soft, 10 kPa), total concentration of norbornene groups (28 × 10^−3^ m) in interstitial matrix, and amount of consumed norbornene groups (86%) in interstitial matrix were kept consistent across groups (Figure S10a, Supporting Information). The behavior of granular hydrogels under compressive loading was investigated (Figure 6b). The use of a guest–host interstitial matrix resulted in a significant increase in compressive modulus, failure strain, and failure stress compared to covalent interstitial matrices. The increases in failure properties were also true for granular hydrogel composites fabricated with medium and stiff microgels (Figure S11, Supporting Information). These increased failure mechanics likely arise from the adaptability of the guest–host network, as guest–host bonds can break and reform during compressive loading to increase the failure properties. Further, we demonstrate that the introduction of a guest–host interstitial matrix does not impact the interstitial space (%) in the granular hydrogel composite system (Figure 6c).

Importantly, the guest–host interstitial matrix significantly enhanced failure properties in granular hydrogel composites, despite having a significantly lower bulk hydrogel compressive modulus (50 kPa) compared to the covalent control (120 kPa). This further highlights the tunability of the granular hydrogel composite system, as the types of crosslinking chemistry within both phases can be adjusted to increase the range of possible mechanical properties.

### 2.7 Introducing Hydrolytically Degradable Microgels into Granular Hydrogel Composites

**Figure 7:**
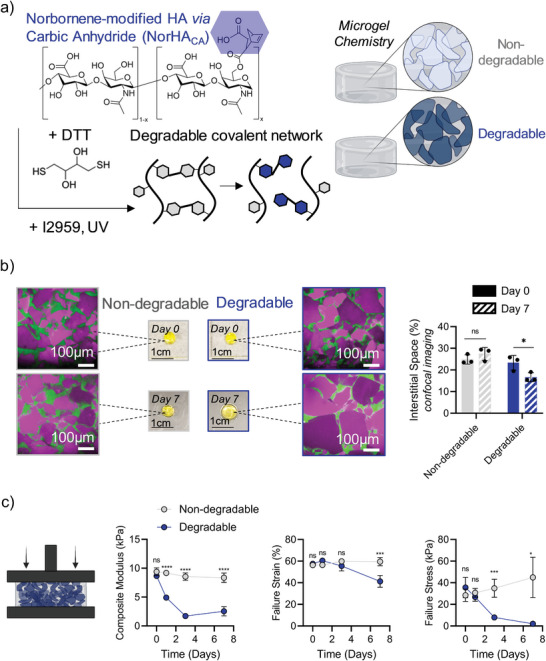
Influence of microgel degradability on granular hydrogel composite properties. Interstitial matrix modulus (120 kPa), total concentration of norbornene groups (8 × 10^−3^ m) in microgels, and total consumption of norbornene groups (50%) in microgels kept consistent across granular hydrogel composites. a) Chemical structure of HA modified with norbornene via carbic anhydride (NorHA_CA_), and combination with dithiothreitol (DTT), Irgacure 2959 (I2959), and UV light to form a photocrosslinkable, degradable network (left). Schematic of granular hydrogel composites with either nondegradable or degradable microgels (right). b) Macroscopic images (scale bar = 1 cm) of nondegradable and degradable granular hydrogel composites with representative confocal slices (scale bar = 100 µm) depicting granular hydrogel composite structures after 7 days (microgels: pink, interstitial space: green) (left), and quantified interstitial space (%) as a function of microgel degradability at Day 0 and Day 7 (right, *n* = 3). c) Quantified compressive moduli (left), failure strain (center), and failure stress (right) of degradable (blue) and nondegradable (grey) granular hydrogel composites over 7 days (*n* = 4). Statistical analysis performed using a one‐way ANOVA. ns = no significance, *p < 0.05, ***p < 0.001, ****p < 0.0001.

A major advantage of the granular hydrogel composite system is the ability to fabricate biomaterials with compartmentalized functionalities, such as degradation profiles, which can allow for temporal programming of mechanical properties and internal microstructure. Degradable granular hydrogels have been explored for applications such as promoting cell infiltration and controlled release of therapeutics.^[^ ^44^, ^51^, ^67^, ^68^ ^]^ In this study, soft hydrolytically degradable microgels were fabricated from norbornene‐modified HA synthesized via carbic anhydride (NorHA_CA_, ≈30% degree of modification [Figure S12, Supporting Information]) and combined with a nondegradable interstitial matrix to form a degradable granular hydrogel composite (**Figure** **7**a). The total concentration and degree of consumption of norbornene groups were kept consistent between degradable microgels (NorHA_CA_) and nondegradable microgels (NorHA) (Figure S13a, Supporting Information). Similar to NorHA, NorHA_CA_ macromer can form hydrogels in the presence of light, DTT (crosslinker) and I2959 (Figure S13b, Supporting Information). Unlike NorHA, where norbornene modification is achieved by conjugation of norbornene carboxylic acid, the norbornene group in NorHA_CA_ consists of an additional carboxyl group which results in increased hydrophilicity and enhanced ester hydrolysis, resulting in hydrolytically susceptible hydrogels. The bulk hydrogel properties of the soft degradable hydrogel used in this study are shown in Figure S13c (Supporting Information).

Degradable granular hydrogel composites maintain their granular structure over a period of 7 days (Figure 7b). Interestingly, degradable granular hydrogel composites exhibit a significant decrease in interstitial space (%) over a period of 7 days, due to the swelling of the degradable microgel phase (Figure 7b). This is also confirmed by the macroscopic swelling (30%) of degradable granular hydrogel composites over 7 days (Figure S14a, Supporting Information). In addition, degradation of granular hydrogel composites was assessed via release of uronic acid, a constituent of the HA backbone. Degradable granular hydrogel composites consisting of NorHA_CA_ microgels steadily released uronic acid over a period of 2 weeks, whereas negligible uronic acid release was observed in nondegradable granular hydrogel composites (Figure S14b, Supporting Information). It is important to note that uronic acid release does not reach 100% over 2 weeks, likely due to the stable presence of the nondegradable interstitial matrix. Nondegradable granular hydrogel composites exhibited limited degradation and swelling during this period.

Initially, there were no significant differences between the compressive modulus, failure strain, and failure stress of degradable and nondegradable granular hydrogel composites. However, the compressive modulus, failure strain, and failure stress of degradable granular hydrogel composites significantly decreased over a 7‐day period (Figure 7c). Importantly, the compressive modulus decreased by ≈5‐fold, and the failure stress decreased by ≈20‐fold after 7 days. In contrast, nondegradable composites exhibited no change in compressive properties over 7 days. However, it is important to note that, unlike granular hydrogel systems without an interstitial matrix, the degradable granular hydrogel composites maintained geometric stability and microstructural integrity as microgels degraded over 7 days (Figure 7b), likely due to the ability of the nondegradable interstitial matrix to hold the structure together. This further highlights the benefits of utilizing a granular hydrogel composite system.

The introduction of degradable microgels into granular hydrogel composites has many potential benefits. For example, microgel degradation can introduce microscale porosity into the system, which is important for cell infiltration in injectable tissue repair applications as well as cell and spheroid outgrowth for in vitro cell culture applications.^[^ ^44^, ^67^, ^69^ ^]^ In addition, therapeutics can be encapsulated into degradable microgels for programmed release.^[^ ^51^, ^70^ ^]^ In the degradable granular hydrogel composite system explored herein, degradable microgels may be utilized for increased cell invasion and controlled therapeutic release, while the nondegradable interstitial matrix may maintain structural integrity for applications such as long‐term cell culture and 3D printing investigations as well as localized injection into tissues. Further, leveraging the degradable granular hydrogel composite system allows for temporal tunability of swelling and mechanical moduli, further enhancing the range of material properties for biomaterials applications.

## 3 Conclusions

HA granular hydrogel composites were fabricated by combining fragmented microgels with photocrosslinkable interstitial matrices. The compressive moduli of microgels (10–70 kPa) and interstitial matrices (2–120 kPa) were varied, along with interstitial volume fraction (0–30%), to thoroughly understand the impacts of these design features on granular composite material properties. While compressive moduli increased with increasing microgel modulus and interstitial matrix modulus, it was determined that failure properties (i.e., strain and stress) increased by combining softer microgels with stiffer interstitial matrices. Further, the local properties of granular hydrogel composites were investigated by both direct visualization of microgels during compressive loading as well as AFM‐based nanoindentation to map regional mechanics across granular hydrogel composites. In addition, interstitial matrices composed of guest–host crosslinks were shown to significantly increase failure properties under compressive loading compared to covalently crosslinked systems. Lastly, granular hydrogel composites containing hydrolytically degradable microgels were characterized. In conclusion, the work herein provides an in‐depth characterization of how granular hydrogel composite composition influences the material properties of granular hydrogels. The characterization described in this work will directly inform biomaterial design to further granular hydrogels for biomedical applications.

## 4 Experimental Section

### Materials

Sodium hyaluronic acid (HA, MW = 60 kDa) was purchased from Lifecore Biomedical. Mono(6‐mercapto‐6‐deoxy)‐beta‐cyclodextrin (CD‐thiol) was purchased from BOC Sciences. 1‐adamantane acetic acid was purchased from Acros Organics. All other reagents were obtained from Sigma‐Aldrich unless otherwise indicated.

### Chemical Modification of Hyaluronic acid (HA)

Norbornene‐modified HA (NorHA) was prepared as previously described.^[^ ^30^ ^]^ Briefly, HA was first modified with tetrabutylammonium salt (HA‐TBA), then dissolved in anhydrous dimethylsulfoxide (DMSO). Norbornene carboxylic acid, dimethyl aminopyridine (DMAP), and ditertbutyl dicarbonate (Boc2O) were added to DMSO, and the reaction proceeded overnight. NorHA was then dialyzed in deionized water (DI water), lyophilized, and stored at 4 °C until use.

Adamantane‐modified HA (AdHA) was synthesized as previously described.^[^ ^63^ ^]^ Briefly, HA‐TBA was dissolved in anhydrous DMSO. 1‐adamantane acetic acid, DMAP, and Boc2O were added to DMSO, and the reaction proceeded overnight. AdHA was then dialyzed in DI water, lyophilized, and stored at 4 °C until use.

To synthesize norbornene‐modified HA via reaction with carbic anhydride (NorHA_CA_), HA (3.4 g) was first dissolved in deionized water (0.2 L) on ice at 0 °C. Depending on the desired degree of modification, carbic anhydride (8.4 g, 20 equivalents) was then added to the solution and the pH was maintained at 8.5–9.5 throughout the 4–5 h reaction via dropwise addition of sodium hydroxide (1 n, NaOH). The solution was then transferred to dialysis tubing (Spectra Por, 6–8 kDa cutoff) and dialyzed against deionized water for 3 days, frozen at −80 °C, and lyophilized.

Lyophilized polymers were dissolved in deuterium oxide (D_2_O) at a concentration of 10 mg mL^−1^ and analyzed using ^1^H‐NMR (Bruker NEO400) to determine degree of modification. Norbornene degree of modification on NorHA was determined to be 25%, adamantane degree of modification on AdHA was determined to be 20%, norbornene degree of modification on NorHA_CA_ was determined to be 30%.

### Hydrogel Precursor Solutions and Bulk Hydrogel Fabrication

Hydrogel precursor solutions were formulated based on desired compositions, containing HA macromer, crosslinkers, and initiator. Across experiments, hydrogel precursor solution compositions were normalized by total norbornene concentration (mm) and degree of norbornene consumption by thiolated crosslinkers. Across all formulations, the total amount of free norbornene groups remaining on microgels after fabrication was kept consistent at 4 × 10^−3^ m, which allowed subsequent crosslinking with the interstitial matrix. Hydrogels were photocrosslinked via a thiol‐ene reaction with exposure to ultraviolet (UV) light (Omnicure S2000 lamp) for 5 min at an intensity of 10 mW cm^−2^.

### Characterization of Bulk Hydrogels

Hydrogel precursor solution (50 µL) was placed into a cylindrical mold (4.6 mm diameter) and photocrosslinked with UV light (10 mW cm^−2^). Mechanical testing was performed (TA Instruments, DMA Q800) to determine the compressive moduli of the bulk hydrogels. Samples were exposed to a preload force of 0.01 N and then compressed at a rate of 0.5 N min^−1^ until 30% strain. The compressive moduli were calculated as the slope of the stress–strain curve from 10% to 20% strain.

Rheological properties were characterized using an oscillatory shear rheometer (AR2000, TA Instruments) fitted with a 20 mm diameter cone and plate geometry and 27 µm gap. Time sweeps (1% strain, 1 Hz) were performed at room temperature to characterize bulk gelation upon exposure to UV light (Omnicure S2000 lamp) for 5 min at 10 mW cm^−2^.

### Microgel Fabrication

An extrusion fragmentation method was used to fragment microgels, as previously described.^[^ ^36^ ^]^ Hydrogel precursor solution (1 mL) was added to a 3 mL syringe (BD) and exposed to UV light for 5 min. The bulk hydrogel was manually extruded through 18G, 23G, 27G, and 30G needles (McMaster‐Carr), adding excess PBS (2 mL) after extruding through the 18G needle to reduce extrusion forces required. Then, microgels were suspended in pure PBS and centrifuged at 15 000 rpm for 5 min. PBS supernatant was subsequently removed. This washing step was repeated 3 times.

### Microgel Characterization

A trace amount of high molecular weight FITC‐dextran (2 MDa) was added to hydrogel precursor solutions before microgel fabrication. Fluorescence microscopy (Olympus BX51) was used to visualize the microgels, and ImageJ was used to quantify microgel size and shape. Microgel diameters were calculated by treating the area of the microgel as a circle and determining the equivalent circular diameter.

### Granular Hydrogel Composite Formation

To form granular hydrogel composites, suspensions of microgels were first jammed by vacuum‐driven filtration (Steriflip, 0.22 µm pores, Millipore). The volume of the jammed microgels was determined and interstitial matrix precursor solution was then added to the jammed microgels on a volume percent basis (e.g., if 1 mL of jammed microgels were obtained by vacuum jamming [maximum packing], adding 100 µL of interstitial precursor solution would result in 10% interstitial solution added). Interstitial precursor solution was thoroughly mixed into the jammed microgels using a metal spatula. To fabricate a granular hydrogel composite, the mixture of microgels and interstitial matrix precursor solution (i.e., granular hydrogel composite precursor) was then exposed to UV light for 5 min.

### Confocal Imaging and Porosity Characterization

A trace amount of high molecular weight FITC‐dextran (2 MDa) was added to the either the microgels or interstitial matrix precursor solutions before granular hydrogel composite fabrication, as indicated. Crosslinked granular hydrogel composites were loaded into a cylindrical polydimethylsiloxane (PDMS) mold and covered with a glass cover slip. An upright confocal microscope (Leica TCS SP5) with a water immersion objective lens (25×) was used to image the FITC‐dextran distributed within the interstitial matrix of the granular hydrogel composites. Volumetric stacks measuring (500 µm)^3^ were taken at randomly selected regions of interest with a *z*‐spacing of 2 µm between successive stack slices. Interstitial space (%) was analyzed as an average of the values in each *z*‐stack using ImageJ. For confocal imaging studies, an interstitial matrix consisting of 4.4 wt% NorHA and 12 × 10^−3^ m DTT precursor solution was used, unless otherwise specified. IMARIS software was used to reconstruct the 3D structure using the same confocal *z*‐stack raw data sets.

### Rheological Characterization of Granular Hydrogel Composite Precursors

Rheological properties of granular hydrogel composite precursors were assessed using an oscillatory shear rheometer (AR2000, TA Instruments) with a 20 mm parallel steel plate geometry set at a 1 mm gap. The rheological properties were assessed prior to photocrosslinking of the interstitial matrix solution. Strain sweeps (1–250% strain, 1 Hz) were used to assess strain yielding properties. The yield strain was determined as the strain at which *G*′ < 0.9 G′ initial). For flow characterization, the viscosity and shear stress were measured during a continuous shear rate ramp from 0 to 100 s^−1^. For rheological characterization, an interstitial matrix consisting of 4.4 wt% NorHA and 12 × 10^−3^ m DTT precursor solution was used.

### Extrusion Printing of Granular Hydrogel Composites

Hollow cylinder structures (height = 1 cm; diameter = 1 cm; filament width = 2 mm) were printed using a custom‐built 3D FDM printer (Velleman K8200). Slic3r was used to generate G‐code based on a 3D model (Blender 2.91) and to control hardware (Repetier). Granular hydrogel composite precursors were loaded into 3 mL syringes with an 18G needle. Print speed (12 mm s^−1^) and flow rate (15 µL s^−1^) were controlled using Repetier. Height maintenance (%) was determined by measuring the heights (mm) of the printed cylinders and normalizing to granular hydrogels with no interstitial solution. For extrusion printing, an interstitial matrix consisting of 4.4 wt% NorHA and 12 × 10^−3^ m DTT precursor solution was used. When indicated, granular hydrogel composite precursor solution was exposed to UV light (20 mW cm^−2^) during ink deposition to overcome collapse of the structure. The syringe and needle containing the ink were covered in foil to prevent photocrosslinking from occurring in the syringe prior to deposition.

### Compression Testing of Granular Hydrogel Composites

A volume of 50 µL of granular hydrogel composite precursor solution was secured in a cylindrical mold and exposed to UV light for 5 min to fabricate a granular hydrogel composite. For granular hydrogels without interstitial matrix, microgels were vacuum jammed in a solution of excess DTT and I2959 and subsequently post‐crosslinked with UV light for 5 min to form inter‐particle covalent crosslinks. Mechanical testing was performed to determine the compressive moduli, failure strains, and failure stresses of the samples. Granular hydrogel composites were subject to a 0.01 n preload force and compressed until failure at a rate of 0.5 N min^−1^. The compressive moduli were calculated as the slope of the stress–strain curves from 10% to 20% strain.

### Visualizing Microgel Deformation During Compression in Granular Hydrogel Composites

Granular hydrogel composites were fabricated such that 1% of microgels contained fluorescent dye (2 MDa FITC‐dextran). A thin film (1 cm × 1 cm × 1 mm) of granular hydrogel composite was fabricated using photocrosslinking and secured between 2 glass microscope slides with PDMS spacers (1 mm). A custom‐built setup was used to compress the granular hydrogel composite film at a strain rate of 5 mm min^−1^ while observing compression under an epifluorescence microscope. Granular hydrogel composites were compressed such that the films were free to deform in one dimension (*x*), compressed in one dimension (*y*), and confined in one dimension (*z*). Strain rate was controlled by securing the compression platform to a syringe pump.

Total compression (%) was determined by observing clear rupture of the composite on the epifluorescence microscope and using the time to failure, strain rate, and sample dimensions to calculate failure strain. Using the video captured by the microscope, the compression (%) of microgels was measured at initial conditions (i.e., the initial height, *h*_i_) and just prior to failure (i.e., the final height, *h*_f_) using ImageJ. Compression (%) of individual microgels at failure was determined as 100% * (*h*_i_ − *h*_f_)/*h*_i_.

### Atomic Force Microscopy (AFM)‐Based Nanoindentation

AFM‐nanoindentation was performed on select granular hydrogel composites. A volume of 50 µL of granular hydrogel composite precursor solution was secured in a cylindrical mold and exposed to UV light for 5 min to fabricate a granular hydrogel composite. The sample was then permanently attached to a glass slide using super adhesive glue. AFM‐nanoindentation was performed in a (500 µm)^2^ area within the center of the granular hydrogel composite using a colloidal microspherical tip (*R* ≈ 5 µm, nominal *k* ≈ 0.03 N m^−1^, HQ:CSC38/Cr–Au, NanoAndMore) in 1 × PBS, following established procedures.^[^ ^71^ ^]^ Measurements were taken every 50 µm, resulting in a 10 × 10 map with 100 sampling points total. To maintain consistency, care was taken to ensure a constant effective indentation rate (10 µm s^−1^), and the indentation depth was within a small range (200–400 nm) for each indentation. For each indentation location, the effective indentation modulus, *E*_ind_, was calculated by fitting the entire loading portion of each indentation force‐depth curve to the Hertz model,^[^ ^72^ ^]^ assuming a Poisson's ratio of 0.49 for highly swollen hydrogels.^[^ ^73^ ^]^

### Characterizing Granular Hydrogel Composites with Degradable Microgels

Granular hydrogel composites were fabricated with hydrolytically degradable microgels made from NorHA_CA_. Samples were submerged in PBS at 37 °C for the duration of the degradation study. For swelling studies, granular hydrogel composite samples were macroscopically imaged at specified times, and sample diameters were recorded. Swelling was determined by measuring the change (%) in sample diameter from initial conditions. Uronic acid release was determined by collecting supernatant at specified times and determining concentration using a uronic acid assay as previously described.^[^ ^74^ ^]^ To image the granular hydrogel composites using confocal microscopy, microgels were labelled with rhodamine‐BSA, and the interstitial matrix was labelled with FITC‐dextran (2 MDa).

### Statistical Analysis

No preprocessing of data was used in these studies. Data is presented as mean ± standard deviation, unless otherwise indicated. Statistical analysis was conducted in GraphPad Prism 8. Statistical analysis performed using a one‐way ANOVA with a Tukey‐Kramer post hoc comparison, which accommodates for testing groups with both equal and unequal sample sizes. For each study, sample size (*n*) is indicated in the figure caption. *p < 0.05, **p < 0.01, ***p < 0.001, ****p < 0.0001, ns = not significant.

## Conflict of Interest

The authors declare no conflict of interest.

## Supporting information

Supporting Information

Supplemental Video 1

Supplemental Video 2

## Data Availability

The data that support the findings of this study are available from the corresponding author upon reasonable request.
