# Supplementary material for: Influence of Microgel and Interstitial Matrix Compositions on Granular Hydrogel Composite Properties
Source: Adv Sci (Weinh). 2023 Jan 30;10(10):2206117. doi: 10.1002/advs.202206117 (PMC10074081; doi:10.1002/advs.202206117)
Supplement: Supplementary file 1 — Supporting Information [file ADVS-10-2206117-s002.pdf]

Supporting Information

Influence of Microgel and Interstitial Matrix Compositions on Granular Hydrogel Composite Properties

Victoria G. Muir, Shoshana Weintraub, Abhishek P. Dhand, Hooman Fallahi, Lin Han, and Jason A. Burdick

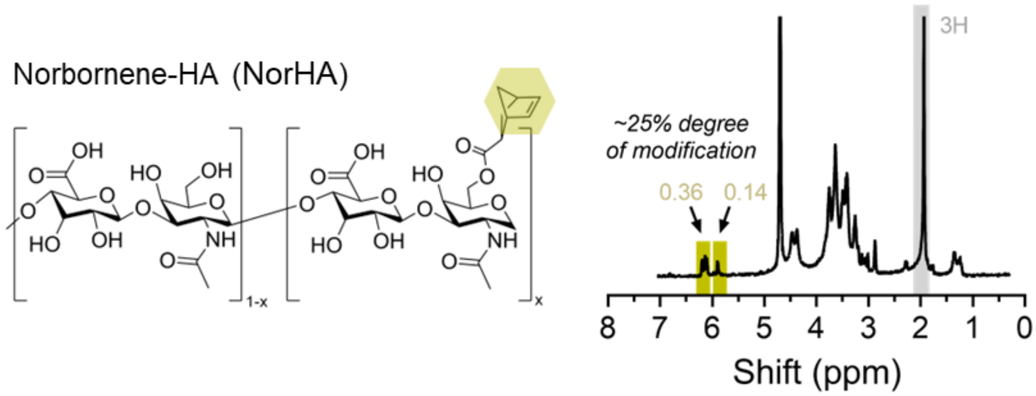

Figure S1. Determining NorHA degree of modification Chemical structure (left) and ^{1}H NMR spectra used to determine degree of modification (right) for HA modified with norbornenes (NorHA). Functional group peaks (gold) were normalized to the HA backbone (grey) and quantified by peak integration. Degree of modification is 25%.

a)

	Hydrogel Precursor Solution Compositions				
Microgel Designation	-	Soft	Med	Stiff	-
DTT (mM)	1	2	4	8	12
Total Norbornene Groups (mM)	6	8	12	20	28
Degree of Norbornene Consumption (%)	33%	50%	67%	80%	86%
Unconsumed Norbornene Groups (mM)	4	4	4	4	4
NorHA (wt.%)	0.95	1.3	1.9	3.2	4.4
I2959 (wt.%)	0.05	0.05	0.05	0.05	0.05
Compressive Modulus (kPa)	2	10	30	70	120

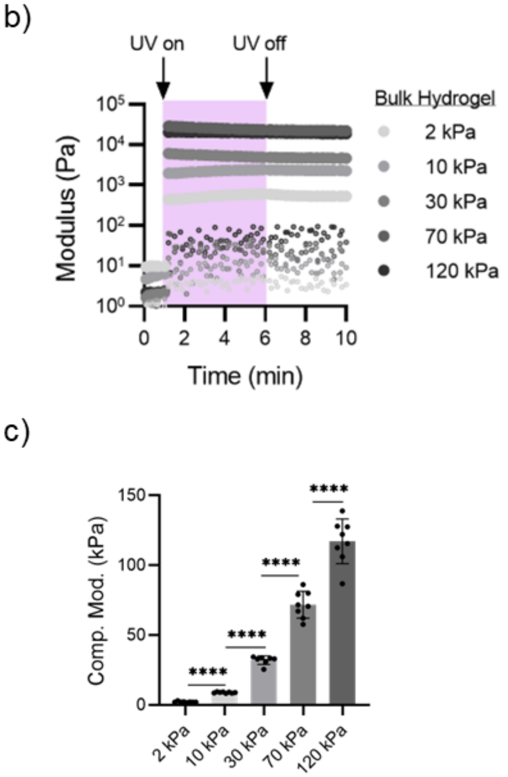

Figure S2. Hydrogel precursor solution compositions and bulk hydrogel mechanics. a) Table of hydrogel precursor compositions used in the fabrication of both microgels and interstitial matrix. b) Oscillatory shear rheology time sweeps showing gelation of bulk hydrogels. Purple indicates exposure to UV light. Storage modulus (G', Pa) represented by closed circles, loss modulus (G'', Pa) represented by open circles. c) Quantified compressive moduli of bulk hydrogel formulations. Statistical analysis performed using a one-way ANOVA. n = 8, ****$p<0.0001$.

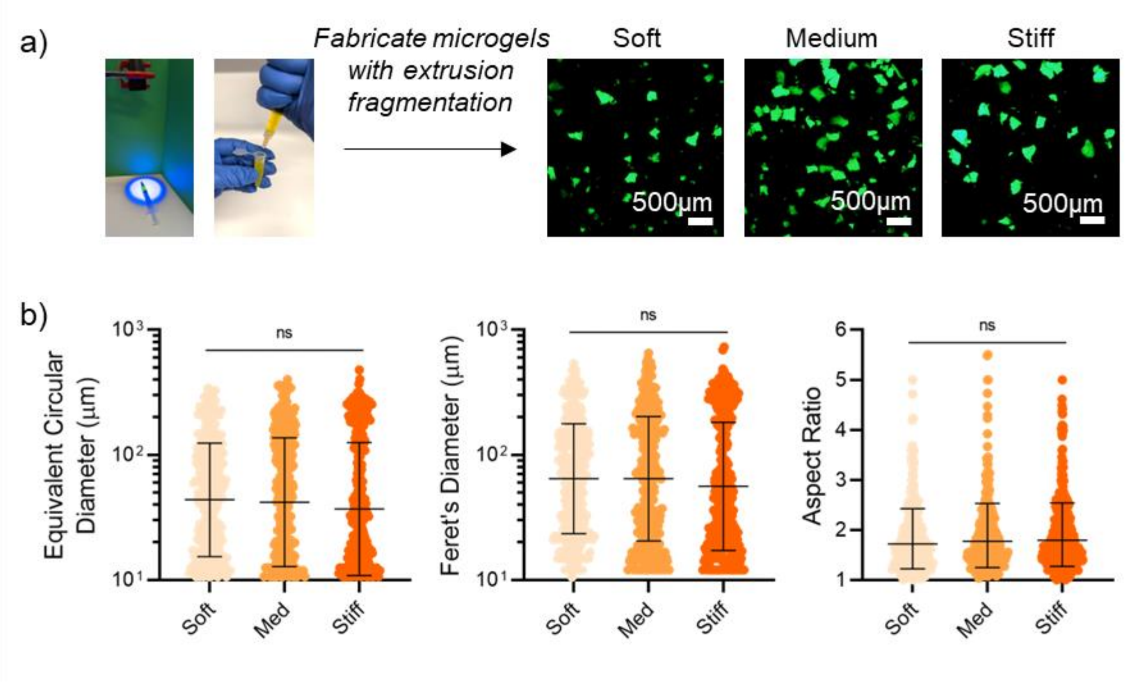

Figure S3. Fabricating microgels using extrusion fragmentation. a) Representative images of extrusion fragmentation process, depicting photocrosslinking in bulk inside of a syringe and extruding through needles to fragment (left). Representative fluorescence microscopy images of fragmented microgels in suspension (right). b) Quantification of equivalent circular diameter, Feret's diameter, and aspect ratio of $n \geq 100$ microgels in a representative batch of extrusion fragmented NorHA microgels (Soft: 10 kPa, Med: 30 kPa, Stiff: 70 kPa). Statistical analysis performed using a one-way ANOVA. n = 300, ns = no significance.

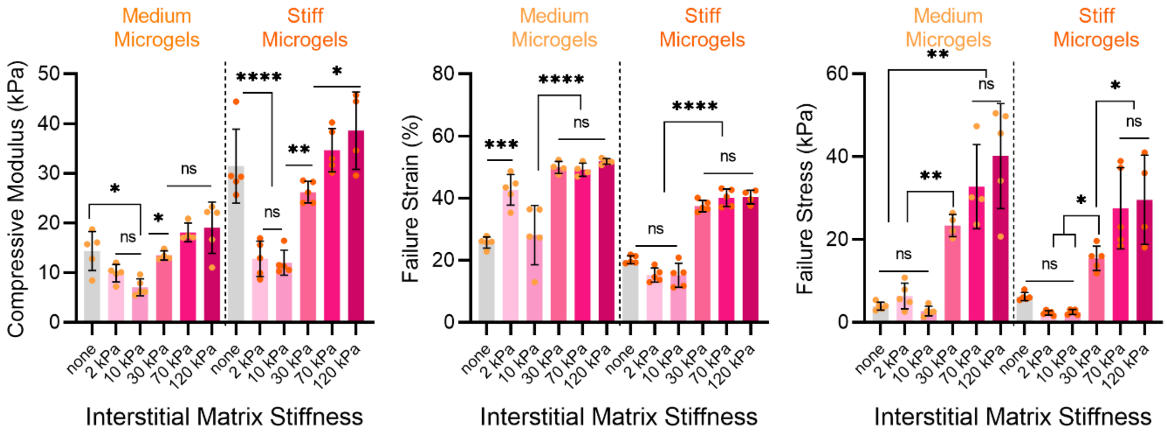

Figure S4. Granular hydrogel composites under bulk compressive loading (medium and stiff microgels. Interstitial matrix volume percent (20%) kept consistent across granular hydrogel composites. Microgel modulus was either Medium (30 kPa) or Stiff (70 kPa). Quantified compressive modulus (left), failure strain (center), and failure stress (right). Statistical analysis performed using a one-way ANOVA. n $\geq$ 4, ns = no significance, *p<0.05, **p<0.01, ***p<0.001, ****p<0.0001.

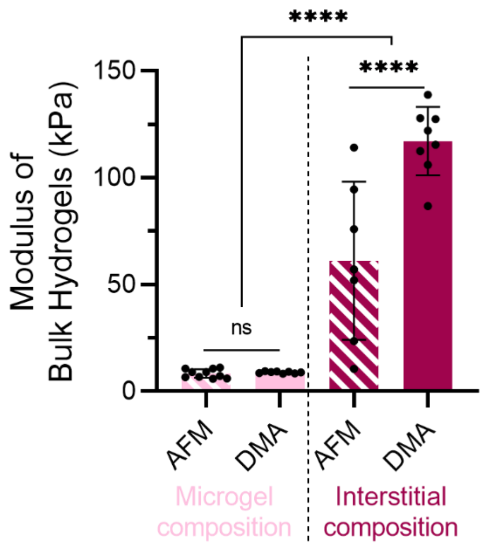

Figure S5. Local moduli of bulk hydrogels determined by atomic force microscopy (AFM). Quantified elastic moduli of bulk hydrogels consisting of soft microgel composition solution (light pink) and 120kPa interstitial matrix composition (dark pink) determined by either AFM or bulk elastic loading using dynamic mechanical analysis (DMA). Statistical analysis performed using a one-way ANOVA. n $\geq$ 6, ns = no significance, ****p<0.0001.

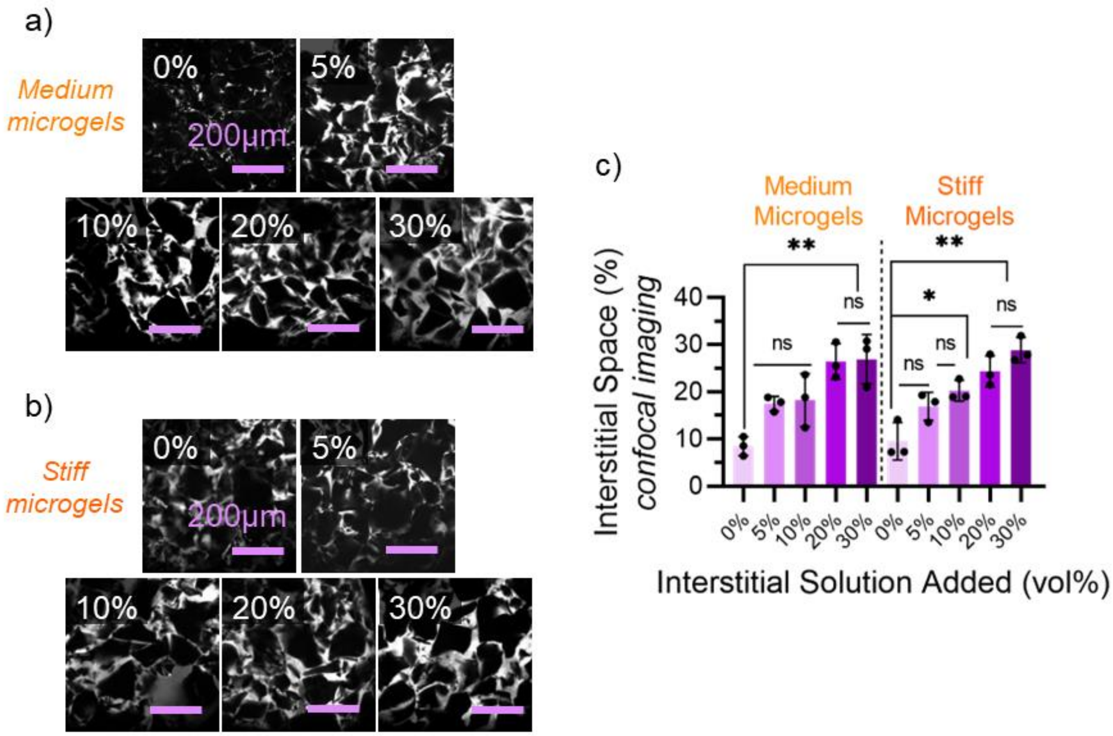

Figure S6. Influence of interstitial matrix/microgel ratio on granular hydrogel composite properties. Interstitial matrix modulus (120 kPa) kept consistent across granular hydrogel composites. a) Representative confocal image slices of granular hydrogel composites showing microgels (black) and pores (white), for medium microgels. Scale bar = 200 µm. b) Representative confocal image slices of granular hydrogel composites showing microgels (black) and pores (white), for stiff microgels. Scale bar = 200 µm. c) Quantified interstitial space (%) as a function of interstitial solution added (vol %). Statistical analysis performed using a one-way ANOVA. n = 3, ns = no significance, *p<0.05, **p<0.01.

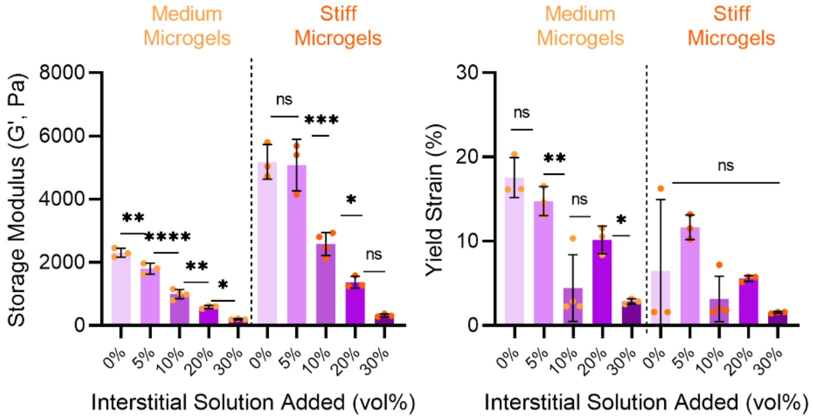

Figure S7. Rheological characterization of granular hydrogel composite precursors. Interstitial matrix modulus (120 kPa) kept consistent across granular hydrogel composites. Quantification of storage moduli (G', Pa, left) and yield strain (%, right) of granular hydrogel composite precursors for various microgel moduli (soft, medium, stiff) and interstitial solution volume percent (0-30%). Statistical analysis performed using a one-way ANOVA. n $\geq$ 3, ns = no significance, *p<0.05, **p<0.01, ***p<0.001, ****p<0.0001.

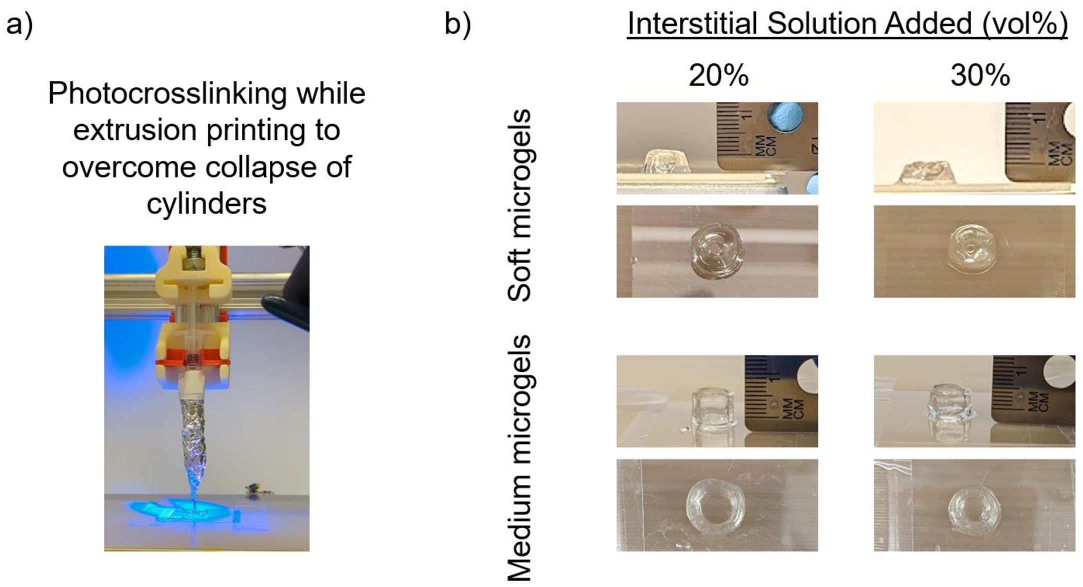

Figure S8. Photocrosslinking during extrusion printing. a) Image showing printed structures being exposed to UV light to induce photocrosslinking during ink deposition. b) Representative images of extrusion printed structures made from granular hydrogel composites (soft or medium microgels; 20 or 30% interstitial solution) that underwent photocrosslinking during ink deposition.

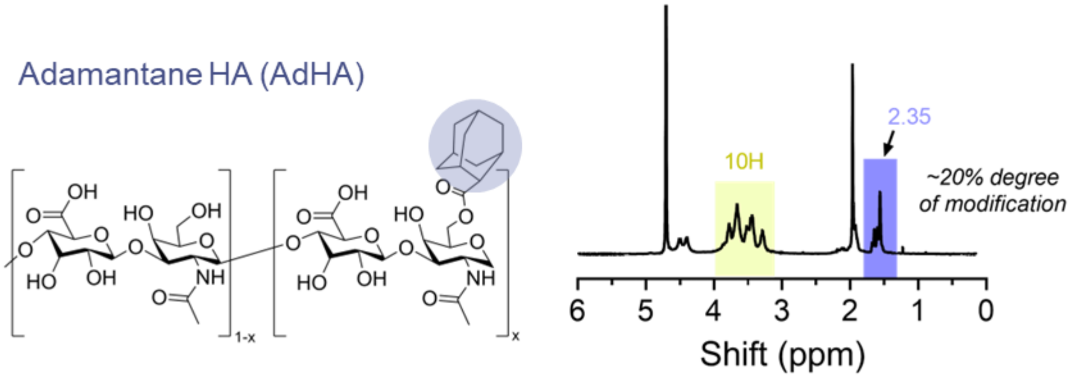

Figure S9. Determining AdHA degree of modification. Chemical structure (left) and ^{1}H NMR spectra used to determine degree of modification (right) for HA modified with adamantane (AdHA). Functional group peaks (purple) were normalized to the HA backbone (yellow) and quantified by peak integration. The degree of adamantane modification was 20%.

a)

	Covalent	Guest-Host
Total Norbornene Groups (mM)	28	28
Degree of Norbornene Consumption (%)	86%	86%
Unconsumed Norbornene Groups (mM)	4	4
NorHA (wt.%)	4.4	4.4
I2959 (wt.%)	0.05	0.05
DTT (mM)	12	-
CD-thiol (mM)	-	24
Total Adamantane Groups (mM)	-	24
AdHA (wt.%)	-	4.8
Compressive Modulus (kPa)	120	50

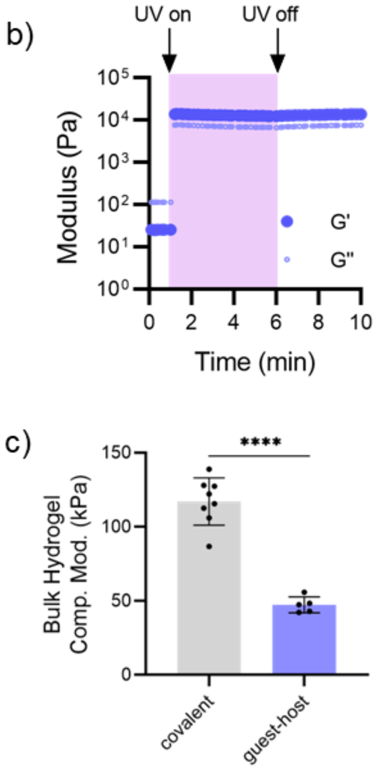

Figure S10. Fabrication of photocrosslinkable guest-host bulk hydrogels and granular hydrogel composites. a) Table of compositions of covalent and guest-host photocrosslinkable hydrogels used as interstitial matrices in granular hydrogel composites. b) Oscillatory shear rheology time sweeps showing gelation of bulk guest-host hydrogels. Purple indicates exposure to UV light. c) Quantified compressive moduli of covalent and guest-host bulk hydrogels used as interstitial matrices in granular hydrogel composites. %). Statistical analysis performed using a one-way ANOVA. $n \geq 5$, ****$p<0.0001$.

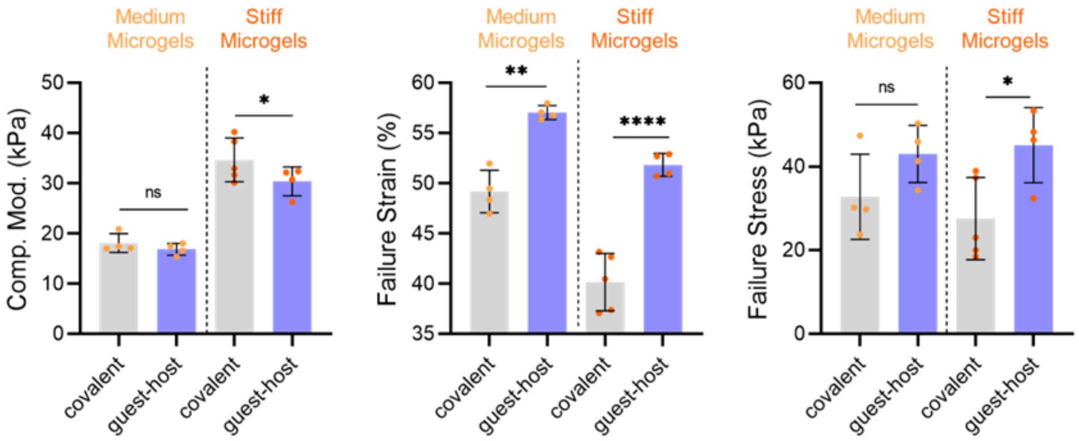

Figure S11. Influence of interstitial matrix crosslinker chemistry on granular hydrogel composite properties. Total concentration of norbornene groups (28 mM) in interstitial matrix and amount of consumed norbornene groups (86%) in interstitial matrix kept consistent across granular hydrogel composites. Microgel modulus was either Medium (30 kPa) or Stiff (70 kPa). Quantified compressive modulus (left), failure strain (center), and failure stress (right). Statistical analysis performed using a one-way ANOVA. n = 4, ns = no significance, *$p<0.05$, **$p<0.01$, ***$p<0.001$, ****$p<0.0001$

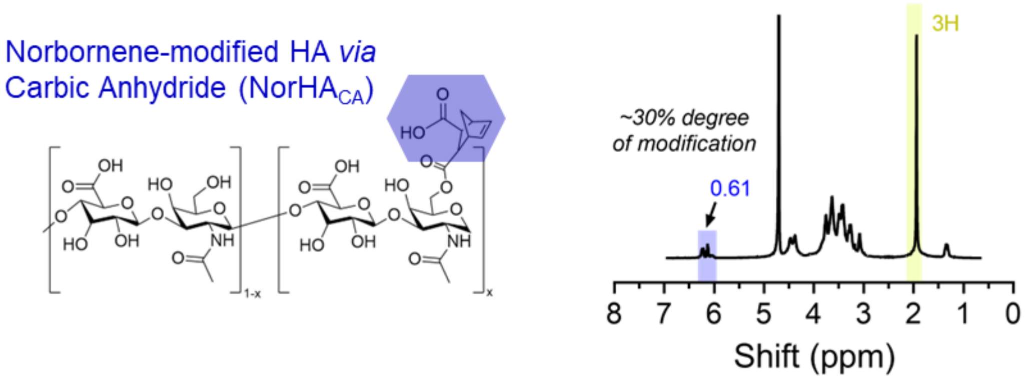

Figure S12. Determining the degree of modification of NorHA$_{CA}$. Chemical structure (left) and ^{1}H NMR spectra used to determine degree of modification (right) for HA modified with norbornene via carbic anhydride route (NorHA$_{CA}$). Functional group peaks (blue) were normalized to the HA backbone (yellow) and quantified by peak integration. Degree of norbornene modification with norbornene groups was 30%.

a)

	Non-degradable	Degradable
Total Norbornene Groups (mM)	8	8
Degree of Norbornene Consumption (%)	50%	50%
Unconsumed Norbornene Groups (mM)	4	4
NorHA degree of modification (%)	25%	30%
NorHA (wt.%)	1.3	1
I2959 (wt.%)	0.05	0.05
DTT (mM)	2	2
Compressive Modulus (kPa)	10	4

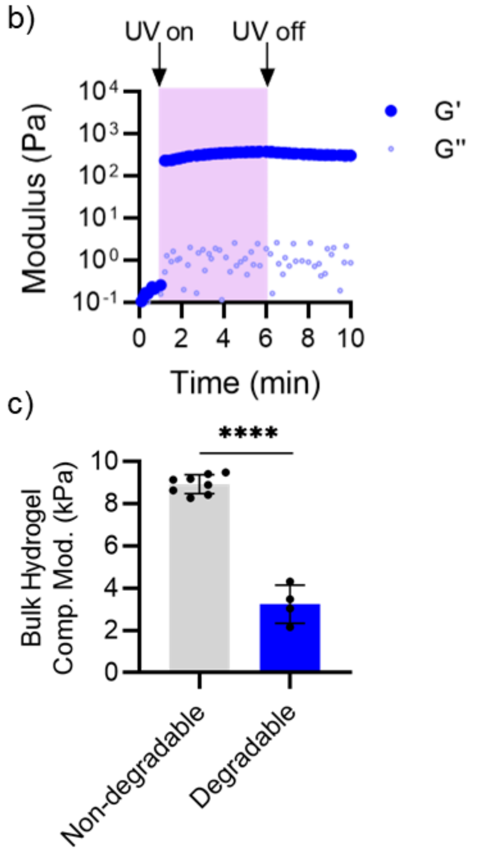

Figure S13. Fabrication of degradable bulk hydrogels and granular hydrogel composites. a) Table of compositions of degradable and non-degradable photocrosslinkable hydrogels used as microgels in granular hydrogel composites. b) Oscillatory shear rheology time sweeps showing gelation of bulk degradable hydrogels. Purple indicates exposure to UV light. c) Quantified compressive moduli of degradable and non-degradable bulk hydrogels used as microgels in granular hydrogel composites. Statistical analysis performed using a one-way ANOVA. $n \geq 4$, ****$p<0.0001$.

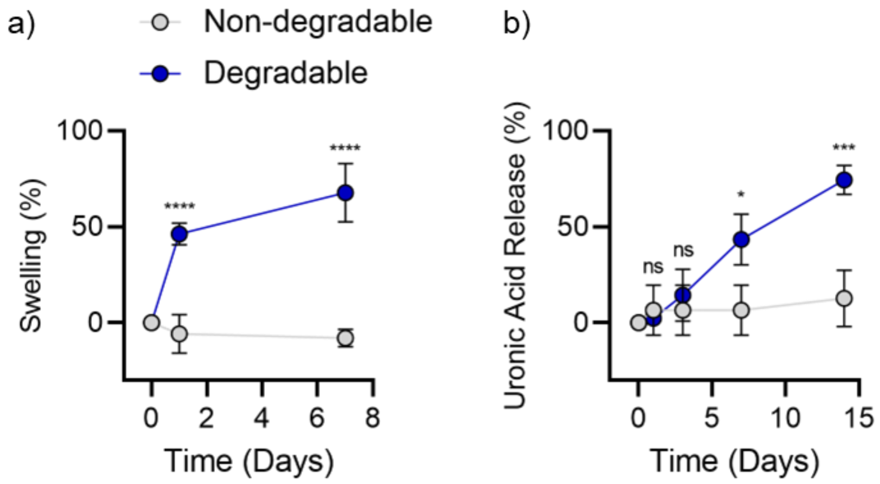

Figure S14. Swelling and uronic acid release from degradable granular hydrogel composites. a) Swelling (%) of degradable (blue) and non-degradable (grey) granular hydrogel composites over time determined from macroscopic images. b) Uronic acid release (%) of degradable (blue) and non-degradable (grey) granular hydrogel composites over time. n = 3, ns = no significance, *p<0.05, ***p<0.001, ****p<0.0001.
